# Confocal and Electron Microscopic Structure of the Cornea from Three Species of Penguin

**DOI:** 10.3390/vision7010004

**Published:** 2023-01-03

**Authors:** Peter W. Hadden, Akilesh Gokul, Satya Amirapu, Ratish Kurian, Charles N. J. McGhee, Jie Zhang

**Affiliations:** 1 Department of Ophthalmology, Faculty of Medical and Health Sciences, University of Auckland, Auckland 1142, New Zealand; 2 Department of Anatomy and Medical Imaging, Faculty of Medical and Health Sciences, University of Auckland, Auckland 1142, New Zealand; 3 Biomedical Imaging Research Unit, Department of Anatomy and Medical Imaging, Faculty of Medical and Health Sciences, University of Auckland, Auckland 1024, New Zealand

**Keywords:** king penguin, *Aptenodytes patagonicus*, little penguin, *Eudyptula minor*, gentoo penguin, *Pygoscelis papua*, confocal microscopy, electron microscopy, cornea

## Abstract

Corneal confocal microscopy has not previously been performed in penguins, despite recognition of its unusually flat shape. To identify features that the penguin shares with other birds and or mammals and those specific to penguins, we undertook confocal microscopic examination of two little (*Eudyptula minor*), four gentoo (*Pygoscelis papua*) and five king (*Aptenodytes patagonicus*) penguin corneas. Transmission electron microscopy was performed on one gentoo and one king penguin, for finer details. Features shared with other higher vertebrates included a five-layered cornea and a similar limbus. Typically avian were a lower density of stromal cells, a more regular arrangement of collagen bands and an absent basal nerve plexus. Features unique to penguins included a flattened superficial epithelium (king penguin), stromal myofibroblasts (all) and an irregular endothelium (little penguin). Other features uniquely identified by confocal microscopy in birds include epithelial and stromal nerves, guttata and stromal imprints on Descemet’s membrane. Transmission electron microscopy identified a lack of wing cells (king penguin), greater posterior collagen lamellae thickness (gentoo penguin) and significantly less interlacing of collagen lamellae in the central cornea (king and gentoo). Most of these unique features are yet to be explained, but some could be adaptations to diving.

## 1. Introduction

The microstructure of the penguin cornea is of great interest because of its amphibious habit, and it has been postulated that its remarkably flat contour is an adaptation to its need to operate in both marine and terrestrial environments [1,2]. However, the shape of the penguin cornea does vary, with the smaller penguins having smaller, more steeply curved corneas [3]. The most recent transmission (TEM) and scanning (SEM) electron microscopic study of the penguin cornea was performed on what was described as a little penguin *Eudyptula minor* [4], although we note that these specimens were obtained in Western Australia while ours came from Auckland, New Zealand and recent evidence suggests that they are different species although of the same genus [5,6,7,8,9]. The cornea of both the former penguin and the Magellanic penguin *Spheniscus magellanicus* have also been the subject of previous electron micrographic studies [2,10,11,12]. However, despite its obvious utility electron microscopy (EM) does have drawbacks, such as the need for fixed tissue, which can cause distortion and tissue shrinkage of up to 30–40% [4].

A more recent development in corneal imaging has been the advent of confocal microscopy. This is usually used as a non-invasive method of examining the living human cornea at the cellular level in healthy and pathological states, without any need for tissue preparation. It is thus a powerful clinical and research tool [13]. It also has been used to examine avian corneas, although not to our knowledge penguins, and a number of differences between mammalian and avian corneas were noted in the one paper that we could identify, specifically ‘the wing cells of the anterior epithelium exhibited much larger, irregularly shaped nuclei…the keratocytes had radial nuclei and were located in a strictly parallel fashion’ [14].

The aim of this study was to use confocal microscopy to examine the microstructure of the cornea of three species of penguin, the king *Aptenodytes patagonicus*, gentoo *Pygoscelis papua* and little penguins. We undertook this study on three different species partly because these three were those most readily accessible in Auckland but, given their vastly different foraging behaviours, known differences in corneal shape and the long period of independent evolution of each species [3,9,15,16,17,18,19,20], we also wished to look for variation within Spheniscidae, and for a possible correlation with size or diving depth. Because TEM of two of these species, namely the king and gentoo, has not previously been published, following confocal microscopy we undertook that examination on one cornea of each to look for any morphological differences between the three species, bar the obvious one of size [4,10,12].

## 2. Materials and Methods

After obtaining permission from the Department of Conservation, New Zealand (68003-DOA, 28 November 2018 and 89983-DOA, 27 July 2021), we obtained eyes from two little penguins (L1 and L2), four gentoo penguins (G1, G2, G3 and G4) and four king penguins (K1, K2, K3 and K4) immediately after they were euthanized because of increasing debility due to age or, in the case of L2, was found dead in captivity. In vivo confocal microscopy was not performed due to ethical concerns with removing the animals from their housing in order to subject them to restraint and anaesthesia at another facility for that purpose. The sample size was the result of the number of penguins being euthanized over the study period of three years; little penguins found dead in the wild were not sought because of potential post-mortem changes. The king and gentoo penguins were spending or had spent their life in captivity at SEA LIFE Kelly Tarlton’s Aquarium in Auckland, New Zealand and were descended from penguins living in South Georgia. All little penguins were originally recovered from the wild in the Auckland Region, New Zealand, but were unable to be rereleased due to physical disabilities not involving the eye. The exact age for these penguins was unknown, although all were adult birds. Demographic data is presented in Table 1, including the corneal curvature where known, obtained using either the IOLMaster 700 (Carl Zeiss Meditech Inc., Jena, Germany), Nidek OPD-Scan III (Nidek Co. Ltd., Aichi, Japan) or Pentacam AXL (OCULUS Optikgeräte GmbH, Wetzlar, Germany). The latter data was included in a previous report [3]. All raw data is available in the online open access repository https://doi.org/10.17608/k6.auckland.c.6217640.v1 (Digital Science, London, UK).

**Figure 1:**
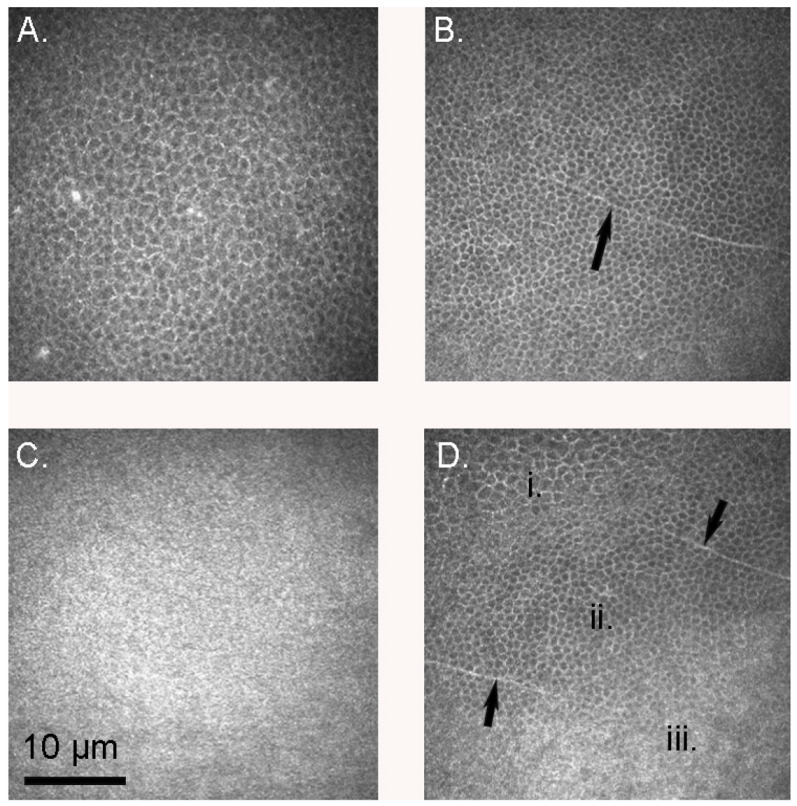
The corneal epithelium of gentoo penguins *Pygoscelis papua*. (**A**) Superficial epithelium (G1, left eye); (**B**) Basal epithelium (G3, left eye). Note the peripheral nerve (arrow); (**C**) Bowman’s membrane (G2, left eye); (**D**). A section (G3, left eye) sliced diagonally to show the superficial epithelium (**i**), basal epithelium (**ii**) and Bowman’s membrane (**iii**). Note how the superficial cells appeared larger in cross section than their basal counterparts, and thus less dense. Arrows indicate peripheral nerves.

All eyes were enucleated following euthanasia. Some were transported directly to the laboratory in a plastic container filled only with air (“Fresh” in Table 1). Where we anticipated a longer period of time between enucleation and examination, the enucleated eyes were stored either in normal saline, Optisol culture medium or air (i.e., without a storage medium) and refrigerated at 4 °C. Confocal microscopy was performed on the cornea using the Heidelberg Retina Tomograph III (HRTIII) Rostock Corneal Module (RCM) (Heidelberg Engineering GmBH, Heidelberg, Germany). The HRTIII is a laser scanning in vivo confocal microscope (IVCM), utilising a coherent Helium Neon diode laser (670 nm). The HRTIII also has a coupled CCD camera lateral to the IVCM, allowing the operator to monitor the position of the HRTIII relative to the cornea. With the 60× objective water immersion lens with a numerical aperture of 0.9 (Olympus, Tokyo, Japan) the IVCM images that are produced are 400 × 400 μm in dimension with a digital image size of 384 × 384 pixels (1.04 μm/pixel), a lateral resolution of 2 μm and optical section thickness of 4 μm. The applanating cap of the HRTIII must applanate (be in contact with) the cornea in order to image the corneal microstructure. Once applanation is achieved, the depth at which the optical section is produced is altered by adjusting the focusing head of the IVCM. As the device is an IVCM, the eye and lens are not immersed in water, instead, Viscotears (Carbomer 980, 0.2%, Novartis, Macquarie Park, Australia) was applied to the eye and used as a coupling agent between the applanating lens cap and the cornea. Eyes were held in place by hand which in turn were resting on the chin rest of the device to maintain a stable position during image capture The full thickness of the central cornea was scanned using the device’s “section mode”. The section mode enables instantaneous imaging of a single area of the cornea at a desired depth. Images were captured continuously to ensure at least three compression free images of each layer were obtained. All images were reviewed and analysed by an experienced examiner (AG) and only compression free images were utilised. Measurements were performed using the proprietary RCM software and cells were counted only if they were readily identifiable rather than a smudge, the implication being that they were in the same plane. Because of variable cellular density (Figure 1B), three different images were counted at each layer of each cornea that was examined, the layers being superficial epithelium, basal epithelium, anterior, mid and posterior stroma as well as the endothelium.

Following confocal microscopy, one piece each of the central and limbal cornea from G4 (right eye) and K5 (left eye) was removed using a Bard-Parker stainless steel size 11 blade and fixed in 2% glutaraldehyde in phosphate-buffered saline overnight. The specimen was then osmicated and dehydrated before embedding in epoxy resin. The resin block was polymerised overnight in a 60 °C oven before being cut and mounted onto copper grids for imaging using a Tecnai™ G^2^ Spirit Twin transmission electron microscope.

**Figure A1:**
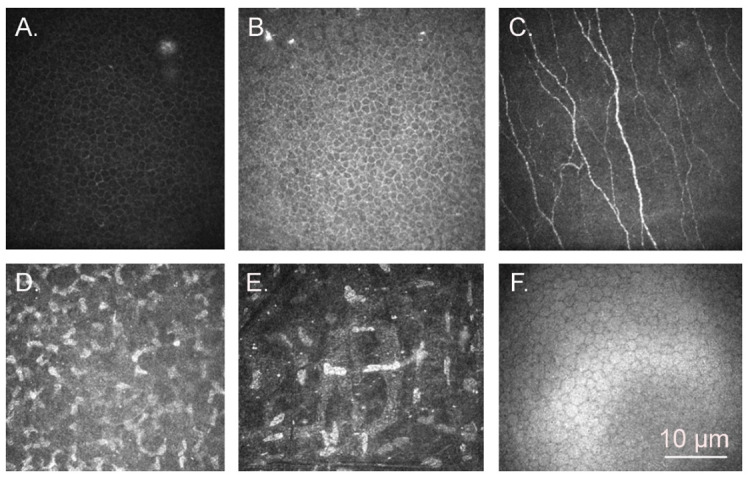
The cornea of a 38-year-old human (*Homo sapiens*) female, as imaged by confocal microscopy. (**A**) Superficial epithelium; (**B**) basal epithelium; (**C**) sub-basal nerve plexus in Bowman’s layer; (**D**) anterior stroma; (**E**) posterior stroma; (**F**) endothelium.

All images were also compared to those of healthy human corneas (Figure A1) [13,21,22].

Statistical analysis: The average and standard deviation of cellular density were calculated for each penguin species at each layer of the cornea. Differences in stromal cell densities between the anterior, mid and posterior stroma of each species, and between cellular densities at the same layer in each of the three species examined, were analysed using one-way ANOVA. If the *p*-value of the homogeneity of variance test, based on the mean, was >0.05, we used Tukey’s post hoc analysis, otherwise we used Games-Howell post hoc analysis. Where only two data sets were able to be compared, a Shapiro–Wilk test was run to determine if a deviation from normal distribution could be detected (the Kolmogorov–Smirnov calculation was unable to be performed due to the small sample sizes) and then an Independent-samples 2-tailed *t* test was performed to look for significant differences.

## 3. Results

On gross inspection, to an experienced corneal surgeon (CNJM), the corneas appeared thinner, more transparent and more easily deformable than the human cornea. The size of the cornea corresponded to the size of the animal [23], the king penguin having the largest cornea and the little penguin the smallest.

### 3.1. Confocal Microscopy

Confocal microscopy allowed for the determination of cellular density in each layer of the cornea, the results of which are presented in Table 2. Within the same species, the basal epithelial cell density was between 1.5 and 3.5 times higher than that of the superficial epithelium, as one would expect from the appearance on confocal microscopy (Figure 1D). This difference was statistically significant in both the little and gentoo penguins (one-tailed *t*-test *p* = 0.04 in the little penguin and 0.024 in the gentoo penguin, assuming normal variances; *p* = 0.038 in the latter if not; Kolmogorov–Smirnov calculations were unable to be performed due to small sample sizes); only one king penguin epithelium was able to be examined and thus this could not be analysed statistically. All penguin corneas had a higher basal epithelial cell density than do human corneas. Morphologically, epithelial cells appeared similar to those of humans, and occasionally a nerve fibre was visible in the basal epithelium (Figure 1). The mammalian sub-basal nerve plexus was absent and Bowman’s layer appeared smooth and amorphous (Figure 1C).

**Figure 2:**
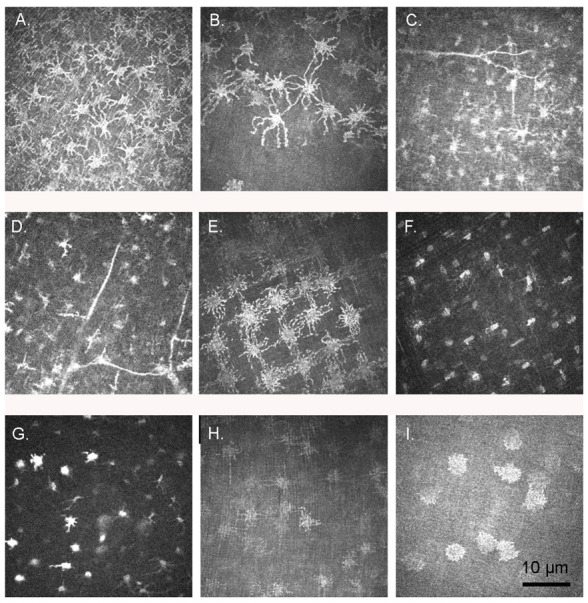
Stromal cells. (**A**) Anterior stroma, little penguin *Eudyptula minor* L2, right eye; (**B**) Anterior stroma, king penguin *Aptenodytes patagonicus* K4, left eye, with clear variation in cell density. In both (**A**,**B**) extensive processes were visible from all stromal cells. These processes were hyperreflective and obscured the nucleus. (**C**) Anterior stroma, little penguin L1, right eye; (**D**). Anterior stroma, little penguin L1, right eye. Note the dichotomous branching of the nerves in (**C**,**D**), which also appeared to touch some of the stromal cells. In (**D**), the branches left at an angle of approximately 90° while in (**C**) the branching angles were more acute; (**E**) Mid stroma, king penguin K3, left eye; (**F**) Mid stroma, king penguin K3, left eye. A lattice like-arrangement of keratocytes was discernible. However, the stromal cells here lacked the extensive processes visible in (**E**); (**G**) Mid stroma, little penguin L1 Similarly thin processes extended from some but not all cells and were shorter than those in (**A**) thru (**E**); (**H**) Posterior stroma, little penguin L2, right eye, again with a lattice-like arrangement of cell bodies and, in this case, processes; (**I**) Posterior stroma, gentoo penguin *Pygoscelis papua* G2, right eye. The processes were shorter and thicker in this section than in some other sections of the same and other penguins.

With regard to the stroma (Figure 2), there were striking differences in keratocyte morphology from the human, with many sections exhibiting larger, rounder cells with multiple hyperreflective cell processes, often linking adjacent cells (Figure 2A–E). The nucleus in such cells was not separately identifiable. The most prominent such cells were in the cornea of L2, which had spent 72 h refrigerated prior to imaging, but they were also seen in others, including K3, which was imaged between 3 and 4 h after euthanasia. However, in other sections some or all of the cells appeared morphologically similar to human keratocytes, with an identifiable nucleus surrounded by a halo of cytoplasm (Figure 2F,G). The distribution of cells also tended towards a regular lattice arrangement, particularly in the posterior stroma (Figure 2E,H), although the density of stromal cells was uneven in some sections (Figure 2B). Although in all species the anterior stromal count was higher than the mid or posterior stroma, the difference did not reach significance in any. The gentoo penguin tended towards a lower cell density in all layers of the cornea except the superficial epithelium and endothelium, a difference which was often significant (Table 2). Dichotomous branching of stromal nerves could also be seen, occurring usually at an angle of 90° (Figure 2D).

**Figure 3:**
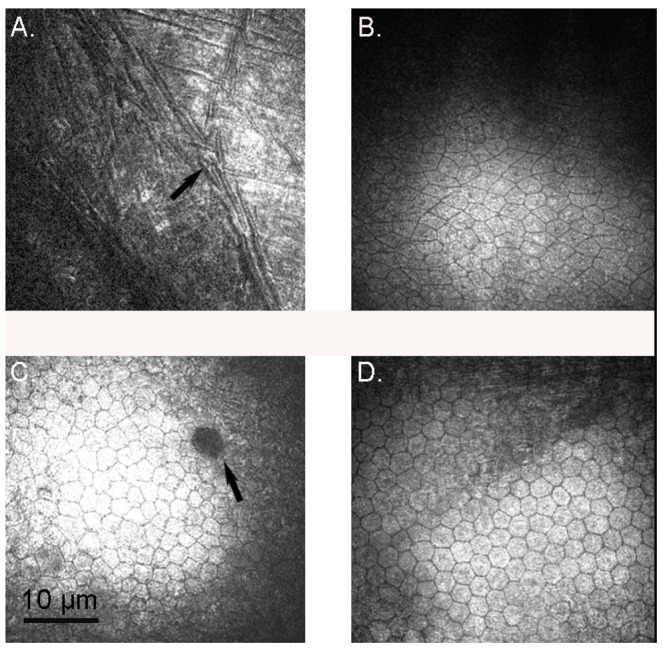
Descemet’s membrane and the corneal endothelium. (**A**) Descemet’s membrane, king penguin *Aptenodytes patagonicus* K2, left eye. The imprint of stromal morphology, either stromal cells or collagen bands, adjacent to the membrane was clearly visible (arrow); (**B**) Corneal endothelium, little penguin *Eudyptula minor* L1, left eye. Note the non-hexagonal nature of the cells; (**C**) Corneal endothelium, gentoo penguin *Pygoscelis papua* G2, left eye. The cells of this 26-year-old penguin were more regular and some are hexagonal, but many were not. There was a dark spot (arrow) which resembled human cornea guttata, more common with increasing age; (**D**) Corneal endothelium, king penguin *Aptenodytes patagonicus* K2, right eye. This 26-year-old penguin had an endothelium with a regular hexagonal pattern that was indistinguishable from the human.

Descemet’s membrane and the endothelium could only be imaged on some specimens, because of technical difficulties obtaining such an image. Descemet’s appeared similar to that in humans, with the imprint of stromal cells presumably immediately adjacent to it clearly visible (Figure 3A). The endothelial cells in the king penguins had the hexagonal array observed in humans [26]; this shape was less obvious in the gentoo and almost no little penguin cells were either hexagonal or symmetric (Figure 3). There was no significant difference in endothelial cell density between species.

### 3.2. Transmission Electron Microscopy (TEM)

**Figure 4:**
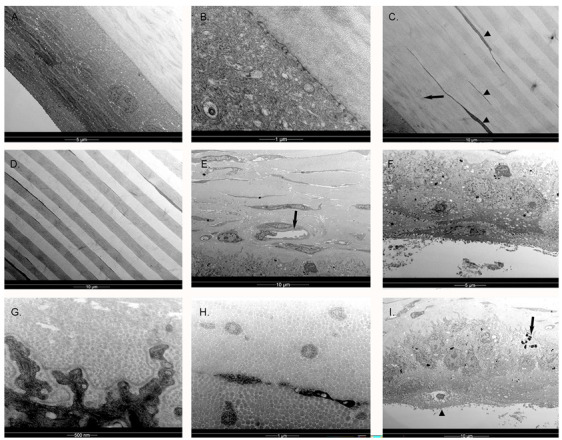
The left cornea of king penguin *Aptenodytes patagonicus* K2, examined by transmission electron microscopy. (**A**) Central cornea. The more superficial epithelial cells were flatter than the basal layer. An intermediate layer of wing cells as described in the human (*Homo sapiens*) cornea was not apparent centrally and vesicles were not as abundant in the superficial cells as in the human; (**B**) Higher power view, showing small interdigitations at the border between the basal epithelium and Bowman’s membrane in the central cornea. There were no anchoring fibrils or plaques; (**C**) There were interconnecting bands (lamellae, arrow) of collagen fibrils in the area of Bowman’s layer, but below the anterior 10 μm of stroma, these bands only very infrequently interconnected. Each band was approximately the same thickness. The collagen fibrils were aligned at right angles to those in the preceding and succeeding bands. The stromal keratocytes (arrowheads) were extremely thin and predominantly located at borders between bands, although their processes occasionally crossed bands; (**D**) In the mid stroma of the central cornea, the collagen bands were slightly thicker. Interconnections between bands remained rare; (**E**) At the limbus, the bands became more irregular and anastomosed more frequently; capillaries were also seen (arrow), with an endothelial cell and pericyte. No fenestration was visible. However, each band was still orientated either perpendicular or parallel to the plane of section. Melanin granules are present in epithelial and stromal cells; (**F**) The limbal corneal epithelium appeared less flattened superficially and had more definite wing cells immediately superficial to the basal layer, similar to that described in the little penguin (*Eudyptula minor)* and human. Multiple desmosomes interconnect all cells. A defined Bowman’s layer was no longer present; (**G**) High powered view of the limbal basal epithelium, showing more complex epithelial-stromal interdigitations in the peripheral cornea than centrally. An electron-dense granular basement membrane was present; (**H**) The collagen fibrils in the limbal stroma could be over 100 nm in diameter, although many wee smaller; (**I**) The equivalent of the epithelial rete peg of the human, forming part of the limbal Palisade of Vogt. Note the melanin granules (arrow) and more squamous superficial cells (arrowhead).

**Figure 5:**
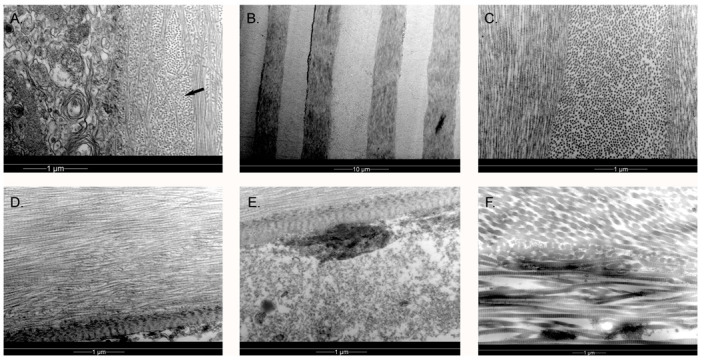
The right cornea of the gentoo penguin *Pygoscelis papua* G4, examined by transmission electron microscopy. (**A**) Anterior central cornea, showing extensive interdigitation between the collagen fibrils in Bowmans’s layer (arrow). Unlike in the human *Homo sapiens*, the collagen fibrils of acellular Bowman’s layer did not appear much thinner than those of the stroma. The orientation of collagen fibres was either perpendicular to or parallel with the plane of section rather than in all directions. The epithelium in this specimen has been affected by post-mortem changes; (**B**) Low magnification, central cornea; (**C**) High magnification, central stroma. Much more regular, thicker and orthogonal layers were visible in the mid and posterior stroma with thin keratocytes at the borders between bands, very similar to the king penguin *Aptenodytes patagonicus*; (**D**) More peripheral posterior stroma and Descemet’s membrane, showing a slightly more irregular structure, especially close to Descemet’s membrane; (**E**) Higher powered view of Descemet’s membrane, with a small remnant of endothelium attached. Note the electron-dense “nodes” forming faint stripes, thinner than the human but similar in thickness to the little penguin *Eudyptula minor*; (**F**) Peripheral stromal collagen fibrils were still organised into bands which are at 90° to their neighbours, although somewhat less regular in thickness and outline. The collagen fibrils were banded, as in the human, with variation in size. The larger fibrils were up to 90 nm in diameter.

TEM of both king and gentoo penguin corneas revealed a structure similar to that described in the little penguin [4,12]. Descemet’s membrane was absent from the king penguin cornea and the endothelium was absent from both, likely because of manipulation and delay prior to processing for electron microscopy, as confocal microscopy was performed prior to such processing. There were also degenerative changes visible in the gentoo penguin epithelium. Hence, we were only able to examine the epithelium, Bowman’s membrane, stroma and, in the gentoo penguin, Descemet’s membrane. The corneal epithelium featured micro-projections on the surface and the more superficial the epithelial cell the more flattened it was, as previously described in the little penguin [4,12]. The peripheral corneal epithelium appeared similar to that described by Collin & Collin [12] in the little penguin; however, centrally, the flattening of the superficial epithelium appeared more extreme in the king (Figure 4A). The corneal epithelium interdigitated with Bowman’s layer (Figure 4B) rather than having specific anchoring fibrils or plaques and these interdigitations became more convoluted towards the limbus. Below the epithelium was an acellular, 10 μm thick Bowman’s layer of interdigitating collagen fibres that were arranged in irregularly branching bundles of fibres, but orientated only either parallel to or perpendicular to the plane of section, i.e., at 90° to each other (Figure 5C). Deep to this were alternating bands of parallel fibres (lamellae), each band being orientated at right angles to its neighbour, and increasing in thickness in the middle and posterior stroma to approximately 5 μm (gentoo) and 2.7 μm (king) in thickness. Anastomoses occurred between lamellae in the anterior stroma but very infrequently in the mid and posterior stroma. Each collagen fibril was similarly sized throughout the central corneal stroma, with an approximate diameter of 24–27 nm (gentoo) and 17–23 nm (king). The centre-to-centre distance of collagen fibres in the central mid stroma was approximately 40 nm in both species of penguin, although it was somewhat variable. Keratocytes were orientated between these bands and were very flattened, although instances were observed of these cells crossing a band. Towards the limbus the arrangement of corneal lamellae became less regular. Collagen fibres in the periphery cornea were observed to have a 60 nm periodic banding, similar to humans [22]. Many of the largest fibrils were 50 nm in diameter, but in some places fibrils over 100 nm (king) and 90 nm (gentoo) in diameter were seen. The keratocytes became plumper and more irregularly scattered in the periphery. Structures resembling the rete epithelial pegs of the human Palisades of Vogt were observed near the limbus (Figure 4G) and melanin granules were present in both peripheral epithelial and stromal cells; an arrangement very similar to the human limbus, which is responsible for the production of new corneal epithelial cells [22].

## 4. Discussion

The penguin cornea has several features that are typically avian, including a lower stromal cell density and a more regular arrangement of stromal collagen bands than in mammals [27]. Features that have not been described previously on confocal microscopy in birds include epithelial and stromal nerves, guttata, processes extending from the stromal cells which may represent myofibroblasts, stromal imprints on Descemet’s membrane and an unusually irregular endothelium, particularly in the little penguin. There are also several unusual findings on TEM, including lack of wing cells in the central corneal epithelium of the king penguin, a greater thickness of the posterior collagen lamellae in the gentoo penguin than in any other reported bird, and more regular and less branched central corneal collagen bands on TEM in both the king and gentoo penguin stroma than has been demonstrated in the little penguin [4]. Conversely, the limbus appeared very similar to that of humans.

The superficial epithelial density was significantly less than that of the more basal cells because, as is clear on TEM (Figure 4A), the superficial cells were more flattened and thus appear larger in coronal section. This is also a feature of the human and little penguin cornea and therefore likely to be true of most if not all birds and mammals [4,28,29]. However, the amount of flattening of the superficial epithelium in the central cornea of the king penguin appeared much greater than that described in either the little penguin or human, with an absence of “wing” cells [4,22]. More typical wing cells were present more peripherally in the king penguin (compare Figure 4A,F). A basal nerve plexus was absent; a previous confocal microscopic study of other birds did not mention this absence specifically but neither did they positively report being able to image such a plexus, whilst they did image and report that of a cat [14]. We therefore suspect this to be a notable difference between birds and mammals. Bowman’s layer appears to be variably present in vertebrates, being absent from dogs and cats but present in humans and those birds that have been studied, including now little, gentoo and king penguins [4,14,22].

The anterior stromal cell density was greater than that of the mid or posterior stroma, as has been reported in other birds and mammals [27], although this difference was not significant. This may be due to the small number of specimens. However, quite why the gentoo penguins as a group tended (although the difference was not always significant) to have a lower stromal cell and basal epithelial cell density than other penguins we are unsure; advanced age is a possibility, although the king penguins were also generally old. The gentoo penguin corneas had generally spent longer time in the refrigerator also, although that seems hard to credit as a reason for this finding. The cross-hatched or lattice-like arrangement of stromal cells has been previously reported in other birds [14], although in this study this arrangement was not present in all slices and was common in the mid and posterior stroma. The presence of stromal cell processes is interesting. In these cells, it is not possible to distinguish the nucleus as both cell body and cytoplasm are highly reflective. During healing, mammalian fibroblasts send out not dissimilar processes composed of hyper-reflective actin and myosin filaments as they transform into myofibroblasts [30]. Dendritic processes have also been observed in human allograft rejection [26]. We suspect that these processes also contain actin and myosin, as they are sufficiently hyper-reflective to obscure the nucleus. This could be a response to the injury of euthanasia and enucleation, although we think is unlikely as they were seen in K2 and K3, both of which were examined within a few hours of death and such processes in a trans-corneal freezing injury develop over a period of days [30]. We therefore speculate that both fibroblasts and myofibroblasts are always resident in the avian cornea. Like other birds, but unlike mammals, each successive corneal lamella is orientated at 90° to its predecessor on TEM [10,27]. We speculate that the lattice-like arrangement of the stromal cells might reflect the more ordered stromal lamellae. Some authors have also theorised that this ordered arrangement of collagen fibres in birds may lead to less light scattering and facilitate vision in the ultraviolet range [27,31]. The diameter of the central corneal collagen fibrils (17–27 nm) was also similar to that found in other birds [4,27]. We are unsure as to why the posterior corneal lamellae were particularly thick in the gentoo and why there were less connections between lamellae in the central cornea of both king and gentoo penguins than in the little penguin [4]. The posterior cornea of both gentoo and king penguins has also been noted to become more swollen post mortem than the anterior half post-mortem, something not seen in the little penguin [3], and perhaps this difference in cross-connections between lamellae may account for that. However, we do note that both gentoo and king penguins have larger and flatter corneas than little penguins. Moreover, gentoo penguins dive to 150 m, twice as deep as little penguins, and king penguins to 300 m [15,16,32], deeper than any other bird yet studied and, since seawater is slightly compressible at depth, their corneas subject to increased pressure [33]. We therefore wonder if these findings are adaptations to allow their flatter, larger corneas to withstand pressure. In the periphery, some collagen fibrils were up to 90 nm in diameter in the gentoo penguin and over 100 nm in the king penguin, a thickness that exceeds the 40 nm described in humans [22]. Whether this could relate to structural strength we can but speculate. However, since the penguin and human periphery share a similar morphology with regard to rete pegs, we suspect a similar limbal location of dividing corneal epithelial cells in both birds and mammals.

Descemet’s membrane resembles that of the little penguin [4] and, apart from being thinner, the human [22]. However, the corneal endothelium was very irregular in each eye of little penguin L1 compared to that of the king penguin and to that described in in other birds [14]. Unfortunately, the endothelium was not present in L2. The endothelium of the gentoo penguins appeared less irregular although still not as regular as in king (this study) or Magellanic [11] penguins, nor as in humans. Examination of more little penguin endothelia may be rewarding. Despite this, the endothelial cell density appeared surprisingly consistent between the three species and significantly less than that reported using SEM in the Magellanic penguin [11]. Interestingly, another paper that used specular microscopy to calculate the endothelial cell density of the goose found a similar density (2409 cells/mm^2^) to what we found in all three penguins, and much less than that found using SEM in other birds [34,35]. We suggest that it is more accurate to determine corneal cellular density using confocal or specular microscopy than electron microscopy because of shrinkage during fixation required for the latter.

In humans, two types of stromal nerves have been described: thick, straight nerves with dichotomous or trichotamous branching and thinner, tortuous nerves often with a beaded appearance [36]. We were only able to observe dichotomous nerve branching, occurring sometimes at an angle of 90° (Figure 2D) and other times at a more acute angle (Figure 2C).

There are several potential sources of error in this study. Confocal microscopy is usually performed on living tissue with the subject fixing on a target, thus allowing precise identification of the central cornea. In this case, the eyes were obtained from recently euthanised birds, which may have undergone post-mortem changes, and were held in what was thought to be the correct orientation while undergoing this examination. A larger number of specimens may have shown more significant differences in cell densities, particularly in the corneal stroma, both between species and at different depths. As mentioned, measurements taken using TEM may also be affected by distortion and shrinkage occurring during fixation, and the time taken to perform confocal microscopy before fixing the tissue for TEM. Finally, as the gentoo and king penguins had spent their entire lives in captivity they would never have been able to dive to any significant depth. It is possible that this lack of exposure to pressure could have influenced the corneal structure when compared to a wild penguin.

In the future, a comparison of the penguin cornea with that of other marine birds, particularly deep diving birds such as the common murre (common guillemot, *Uria aalge*), which has been recorded to dive to 180 m, not dissimilar to the gentoo and king penguin [37], would help elucidate the importance or otherwise of submersion as a factor behind the unusual corneal features seen in these penguins, particularly because there do appear to be significant differences between the shallower-diving little penguin and its deeper-diving relatives. Confocal microscopic examination of the cornea of other birds would also be helpful in determining if the processes observed on some stromal cells are unique to penguins or are typically avian features, and in future studies of corneal cellular density as opposed to electron microscopy.

## Figures and Tables

**Table 1:** Demographic data of penguins including, where known, the names, identification numbers, age at death, sex, storage of the eye until confocal examination and average corneal curvature (including instrument used), for each penguin. The exact ages of birds recovered from the wild were unknown, but all had passed their first moult and are listed as “adult”. Both eyes from the same penguin were examined unless otherwise noted.

Penguin Species and Identification ^1^	Age (Years)	Sex	Preservation Prior to Examination	Right Corneal Curvature ^2^ (D) and Instrument	Left Corneal Curvature ^2^ (D) and Instrument	Remarks
Little penguins *Eudyptula minor*						
L1 (B80231)	Adult	Unknown	Fresh ^3^	40.48 IOLMaster	40.72 IOLMaster	
L2	Adult	Male	72 h at 4 °C ^4^, in air	40.25 Nidek OPD	40.25 Nidek OPD	
Gentoo penguins *Pygoscelis papua*						
G1 (G140, Horse)	26	Male	36 h at 4 °C ^4^, in normal saline		26.95 Pentacam	Left eye only
G2 (G198, Stanley)	26	Male	36 h at 4 °C ^4^, in normal saline	20.45 Pentacam	22.55 Pentacam	
G3 (G144, Dennis)	26	Male	36 h at 4 °C ^4^, in normal saline		22.8 Pentacam	Left eye only
G4 (G194, Twinkle)	26	Female	24 h at 4 °C ^4^, in air			Right eye used for TEM
King penguins *Aptenodytes* *patagonicus*						
K1 (K055, Eskie)	13	Female	36 h at 4 °C ^4^, in air	20.22 Nidek OPD	20.255 Nidek OPD	
K2 (K201, no name)	26	Male	Fresh ^3^			Left eye used for TEM
K3 (K203, no name)	26	Male	Fresh ^3^			
K4 (K158)	30	Female	48 h at 4 °C ^4^, in Optisol culture medium			

^1^ Identification number and name, where known. ^2^ The average anterior corneal curvature. ^3^ Fresh means the ocular examination was completed within 6 hours of death, without being stored in any medium. ^4^ 4 °C was achieved by storage in a refrigerator.

**Table 2:** Cell densities in different layers of the penguin cornea (mean ± standard error of the mean). The total number of eyes examined in each layer of the cornea is noted in brackets. The gentoo penguin *Pygoscelis papua* tended to have a lower stromal cell density regardless of the depth at which the density was examined; the difference reached statistical significance versus the little penguin *Eudyptula minor* in the anterior and mid-stroma and significantly less than the king penguin *Aptenodytes patagonicus* in the posterior stroma. It also had a significantly less dense basal corneal epithelium than the little penguin. There was no significant difference in superficial epithelial or endothelial cell density between species.

	Little Penguin *Eudyptula minor*	Gentoo penguin *Pygoscelis papua*	King Penguin *Aptenodytes patagonicus*	Homogeneity of Variance Test *p*-Value (Based on Mean) ^1^	*p*-Value One-Way ANOVA ^1^	Significance	Human Cornea [24]
Mean superficial epithelial cell density (cells/mm^2^)	5756 ± 948 (2) ^1^	5725 ± 455 (4)	5320 (1)	-	-	No significant difference (2-tailed *t* test) ^2^	n/a
Mean basal epithelial cell density (cells/mm^2^)	19,795 ± 2837 (3)	9602 ± 2975 (4)	16,180 (1)	-	-	*p* = 0.006 (if normally distributed) or *p* = 0.007 (if not normally distributed) ^2,3^	5823 ± 602
Mean anterior stromal cell density (cells/mm^2^)	447 ± 106 (4)	193 ± 43 (4)	288 ± 93 (8)	0.329	0.004	Little v Gentoo 0.003, Little v King 0.028, Gentoo v King 0.221	786 ± 244
Mean mid-stromal cell density (cells/mm^2^)	341 ± 113(4)	132 ± 72 (4)	231 ± 87 (8)	0.522	0.021	Little v Gentoo 0.016, Little v King 0.156, Gentoo v King 0.220	n/a
Mean posterior stromal cell density (cells/mm^2^)	387 ± 172 (4)	149 ± 30 (3)	265 ± 96 (8)	0.022	0.029	Little v Gentoo 0.133, Little v King 0.456, Gentoo v King 0.029 ^4^	293 ± 35
Mean endothelial cell density (cells/mm^2^)	1873 ± 369 (2)	2189 ± 632 (4)	2001 ± 529 (7)	0.651	0.667	No significant difference	2000–3000 ^5^

^1^ Unable to be performed, as only two data sets had *n* > 1. ^2^ Only the little and gentoo penguins were compared, as only one king penguin cornea was able to be measured. An independent values 2-tailed *t* test was performed. ^3^ The Shapiro–Wilk test could not show that either the little penguin set or the gentoo penguin set deviated from a normal distribution; the Kolmogorov–Smirnov calculation was unable to be performed due to the small sample sizes. ^4^ Games-Howell used as *p*-value of homogeneity of variance < 0.05. ^5^ From Ref. [25].

## Data Availability

All raw data is available in the online open access repository https://doi.org/10.17608/k6.auckland.c.6217640.v1; Digital Science, London, UK).
